# History and Structure of the Fourth Leading Emergency Medical Service in the World; a Review Article

**Published:** 2019-02-12

**Authors:** Khosro Shakeri, Mehdi Jafari, Hamidreza Khankeh, Hesam Seyedin

**Affiliations:** 1 Department of Health in Disasters and Emergencies, School of Health Management and Information Sciences, Iran University of Medical Sciences, Tehran, Iran.; 2 Department of Health Service Management, School of Health Management and Information Sciences, Iran University of Medical Sciences, Tehran, Iran.; 3 Health Management and Economics Research Center, Iran University of Medical Sciences, Tehran, Iran.; 4 Health in Emergency and Disaster Research Center, University of Social Welfare and Rehabilitation Sciences, Tehran, Iran.; 5 Department of Clinical Science and Education, Karolinska Institutet, Stockholm, Sweden.

**Keywords:** Emergency medical services, emergency responders, emergency medical technicians, ambulances, history of medicine; Iran

## Abstract

Over forty-three years have passed since the foundation of pre-hospital emergency care system in Iran. Considering the changes that have taken place in recent years in pre-hospital emergency and limited studies in this area, the present review aimed to describe the history, organizational structure, combination of workforce, dispatch system, medical direction and innovations in the pre-hospital system of Iran. The present paper also discusses the strengths and weaknesses of the current system.

## Introduction:

Iran is a country located in the southwest Asia and in the Middle East with an area of 1,873,995 km^2 ^and 79,926,000 million population (2016), 74.5% of which live in cities (1). The average annual population growth rate was 1.24% in the years 2011-2016. According to the latest divisions made by the end of 2015, Iran consists of 31 provinces, 429 counties, 1057 sections, 2589 villages and 1245 cities (2). According to the World Health Organization's report (2017), Iran is among the upper middle-income countries. Life expectancy in males, females, and overall in both genders is 74.5, 76.6, and 75.5 years, respectively (3, 4). The national GDP growth rate was 3.7% in 2017. In 2016, there were 954 active hospitals in the country which had increased by 6% compared to 2015 and state and non-state hospitals accounted for 80% and 20% of the total hospitals, respectively (2). Healthcare services are provided by the Ministry of Health and Medical Education. One of the most important services is emergency service, including pre-hospital and hospital care. In the following sections, we will review the history and structure of emergency medical services (EMS) in Iran.

**History of EMS establishment in Iran**

At least 17 people were killed and lots were injured following the collapse of the ceiling in one of Mehrabad airport waiting rooms on December 5, 1974. Until that time, there was no pre-designed system to help and transfer patients and the injured to the hospital in such sudden accidents. Therefore, the Ministry of Health and Welfare decided to set up a system that met the urgent medical needs. At the end of the same year, according to the amending budget law in 1973 and the nationwide budget in 1974 approved on February 8, 1975, Note 52, the Healthcare Provider Organization was allowed to establish a center providing emergency medical care. On February 2, 1975, Dr. Asad Aram, deputy director of Health Service of Ministry of Social Welfare assigned Dr. Yousef Shakib Fahimi to carry out affairs related to the Emergency Center of Tehran. Therefore, Dr. Fahimi went on a 20-day trip to the United States of America on March 10, 1975 to study and visit the EMS system of that country. After the EMS system of United States was studied, Tehran's Emergency and Information Center was established on Wednesday, September 17, 1975 in the presence of Amir-Abbas Hoveyda, who was the Prime Minister then, and then operationalized. Considering its solid structure and modern medical equipment, the EMS of Tehran was considered the fourth leading EMS in the world after the US. In order to teach the paramedic course to the emergency medical technicians, the head of Tehran EMS travelled to the US for 21 days on January 25, 1976. Considering the extensive need to develop these services across the country, the draft of the EMS Plan was prepared on July 1, 1978. Gradually, these services were expanded to other provinces of the country. Later on, considering a change in the political system of the country in 1979 and the beginning of the imposed war against Iran in 1981, a long interruption was observed in the initial EMS model. Later, a comprehensive coverage EMS plan was approved in 2000, and then in 2001 it was announced that the previous disorganized system was organized and structured significantly.

**Figure:**
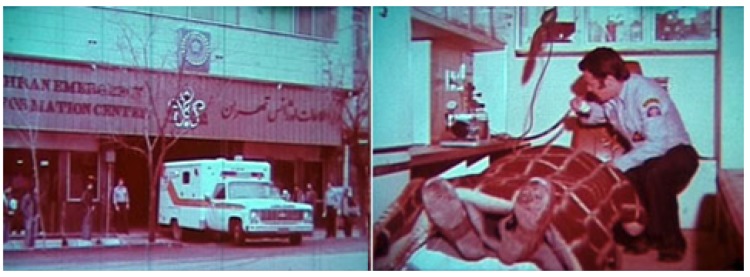
Tehran's Emergency service and Information Center in 1975.

**Structures,** **Funding and Cost**

At the moment, the Iranian EMS system offers free services under the supervision of the Ministry of Health and Medical Education and its main task is to provide emergency medical care to patients and the injured at the accident site and during their transfer to the hospital. The EMS budget is provided by the Ministry of Health from the national annual budget. ''Disaster and Emergency Management Center'' was established in the structure of the Ministry of Health and Medical Education under the supervision of the treatment deputy in 2005. This center was responsible for inter- and trans-sectoral policymaking, planning, organizing, monitoring, and coordinating. The mentioned center operates under the supervision of the Vice-Chancellor of Treatment in all 63 universities of medical sciences in different cities, except for the city of Tehran. The Emergency Center of Tehran (the capital of Iran) is the only center that is not affiliated to any university; it operates directly under the supervision of the Treatment Deputy of Ministry of Health. This structure continued until 2017 when the Statute of the "state emergency organization" was approved by the Government cabinets on January 24, 2018 (5). However, to date, a new organizational chart has not been announced. The global EMS model is often based on Anglo-American or Franco-German models (6). The Anglo-American model was supposed to be implemented in Iran initially, and there was a time that a mix of the two above-mentioned models was applied (7). Currently, it's more like the Anglo-American model. The provided services are often basic life supports (BLS) (8). Nevertheless, advanced measures such as intubation, advanced resuscitation, and use of defibrillator and ventilator are also carried out. Different organizations provide emergency services in different countries, such as municipality, fire department, police, hospitals, private companies, the Red Cross, and volunteers (9, 10). On the other hand, in Iran, Ministry of Health is the only entity authorized to carry out such measures and private companies rarely operate in some centers and towns and only provide human resources. There are private ambulance centers in the country, but they do not play a direct role in the provision of EMS and are more likely to be involved in the inter-hospital transfer and non-emergency cases. Iranian Red Crescent Society also provides basic emergency medical care on some roads and mountainous areas lacking emergency bases, but its main role is to respond to natural disasters. The EMS coverage is 100% at the national level, and all people have access to them by dialing 115. The national distribution of emergency bases in residential areas and roads is based on the population index and time or longitudinal intervals, respectively. According to the fifth development plan, the emergency base-population ratio in urban areas and on roads is as follows: One base with an ambulance for each 20000 population and one base or arrival time of less than 14 minutes per 40 km. Unfortunately, we are far from reaching these standards. Iran has a single tiered-emergency response system.

**Pre-hospital EMS Personnel**

When the EMS system was established in Iran, its human workforce, known as an emergency medical technician (EMT), was recruited after passing a standard theoretical and practical training course. According to existing documents, the paramedic course was supposed to be taught to these technicians based on the Anglo-American model. However, it was forgotten as Iran's political system underwent the momentous changes and the imposed war began. The EMS continued to provide its services in Tehran and across the country for many years at the same level of education. Gradually, with the development of urbanization and population growth, increasing accidents and casualties, increased demand for EMS, and the retirement of old personnel during the years 1998-1999, nurses and the anesthetic and operating room technicians, who were all men, entered this field, which in turn led to a dramatic improvement in the quality of care (11, 12). Considering the increasing demand for services and the need for quantitative and qualitative development of services at the national level, and the shortage of specialized personnel in the pre-hospital field, Associate and Bachelor's degree in this field was included in the curriculum of some universities of medical sciences in 2005 and 2011, respectively. Today, more than 1,000 students with Associate and 500 students with BA degrees in medical emergency graduate from 58 and 33 Iranian universities of medical sciences, respectively each year (13, 14). Physicians attended ambulances for a very short time interval and currently, no general physicians attend the ambulance and most of them do their duties in dispatch centers as medical directors and some as head of emergency centers. Nearly 14,000 people are currently providing EMS as emergency medical technicians including nurses, those with associate and BA degrees in medical emergency and drivers. There are two male technicians in each ambulance, often nurses and medical emergency technicians, who provide EMS to the patients. Drivers rarely attend the ambulances. Human resource limitation is one of the main challenges in this regard (15). In order to respond to the emergency needs of women in Tehran, Shiraz, and Behbahan, special ambulances were launched in 2018 that accommodates two female nurses and a male driver. It is necessary to carry out further research on the patient's satisfaction and the effectiveness of such a service.

**Training**As mentioned earlier, Iranian emergency personnel have university degrees in nursing, anesthesia, and medical emergency. In fact, they are familiar with the basic principles of medicine, treatment and care for the sick and injured. When employed in the emergency system, these people are trained for 72 hours on how to carry out missions and processes by attending the training bases as third persons; and then, they begin their activities officially. They attend optional in-service training throughout the year. One of the national training courses that began in 2016 is the pre-hospital trauma management (PHTM) course and efforts were made to help all technicians complete this course, successfully. In addition, a two-day advanced cardiac life support (ACLS) course was proposed and implemented from the beginning of 2018. Negative points of the field of education include the optional (not compulsory) participation in training classes and weakness of training programs (16, 17). However, it is predicted that all technicians will soon be subject to a scientific evaluation in a nationwide test. Furthermore, the establishment of emergency medicine specialty since 2001 and the presence of its students and graduates in the hospital and pre-hospital services and their participation in the training of EMS personnel led to an improvement in the training process. Challenges in this area include curriculum issues of emergency medical technicians in universities of medical sciences.

**Figure:**
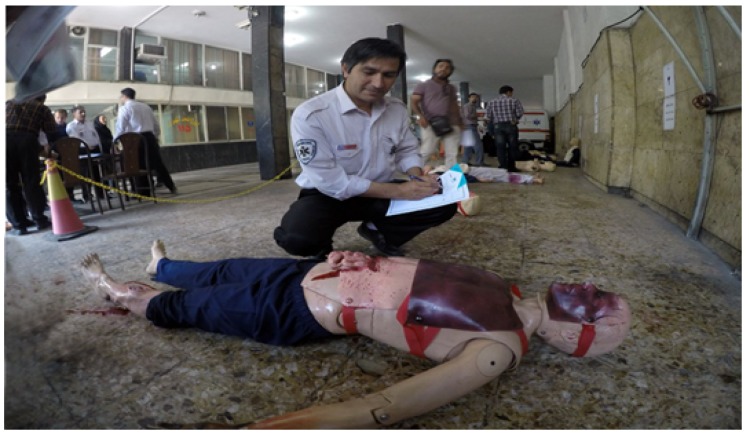
Example of in-service training for EMTs in Tehran emergency center.

**Dispatch System**

The emergency telephone number was 123 at the time of its establishment, which was changed to 115 in 1994. The telephone number was dialed around 48,000 times daily in 2017, out of which 9,000 led to practical missions. It is a toll-free telephone number. The police and fire department telephone numbers are 110 and 125, respectively, each with separate dispatch centers (18). These two organizations do not play a role in providing EMS and do their own duty. There is a dispatch center in each university of medical sciences or provincial center or cities with more than 250,000 population, which is responsible for answering emergency calls and then providing counselling or dispatching and coordinating rescue forces. The message center is responsible for answering calls, providing telephone counseling or dispatching and admitting patients in cities lacking a university or dispatch center (Message Center: In the counties without the necessary conditions to establish a dispatch center and having a population of less than 250,000, the message center has been established and coordinates EMS as an information unit). One of the problems encountered in this area is the fact that there is still no automatic number identification and automatic location identification system in many parts of the country, which will prolong the response time (19). However, we have witnessed improvements in recent years (2018); e.g., a mobile system replaces the wireless system and paper forms in Tehran, and all actions are carried out using mobile applications. A software is used for receiving the patient's characteristics, chief complaints, and the actions taken by the technicians in the ED prior to the arrival of the ambulance to the hospital, which in turn plays a significant role in preparing the ED and the medical team for proper and quick action. It also facilitates analysis of mission statistics. A major challenge includes the lack of the same telephone number (SOS) for emergency rescue services such as EMS, as well as fire and police departments. If implemented, the system will reduce response time and inter-organizational inconsistencies (20, 21).

**Figure:**
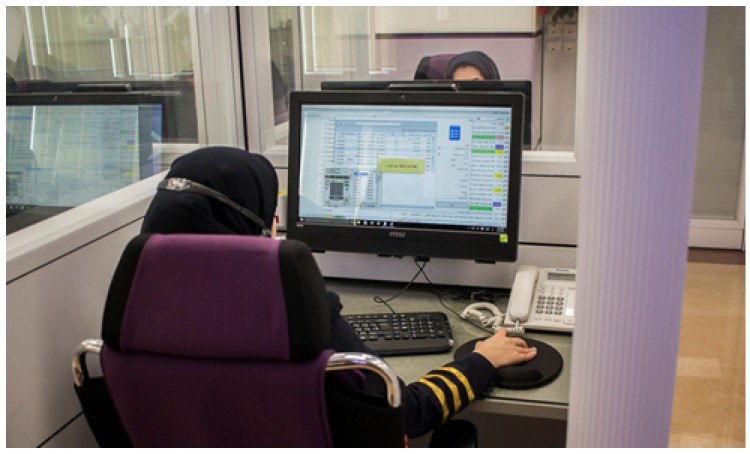
A nurse serving as an Emergency Medical Dispatcher.

**Ambulance and Equipment**

Iran EMS system has witnessed a gradual improvement in its equipment and ambulance type since its establishment. Type-B ambulances are used in the EMS of Iran (22); and since 2005, efforts were made to purchase and distribute Sprinter ambulances in Iran according to its climate. Currently, 5500 sprinter ambulances are active in this system, which has been doubled from 2005 (2880 ambulances). Most ambulances are equipped with a global positioning system (GPS), which facilitates their guidance and observation. The equipment in each ambulance includes long backboard, short backboard or Kendrick extrication device (KED), stair chair, stretcher, cervical collar, splint, automated extracorporeal defibrillator (AED) or defibrillator, ventilator, intubation and airway management equipment (laryngoscope, tracheal tube), pulse-oximeter, oxygen capsule, suction, dressing equipment, and peripheral venipuncture equipment; in Tehran, telecardiology with the capability of taking the 12 leads ECG in the patient's bedside and sending data to the dispatch unit is also available. Ambulance buses are used in order to provide EMS in the event of disaster, traffic accidents, and mass gatherings. There are now 64 ambulance buses, showing a 5-fold increase compared to 2005 (n=12). In order to overcome heavy traffic in metropolitan areas and provide rescue services in the shortest possible time, motorcycle ambulances were used in Tehran and Shiraz in 2005. Twenty-two cities today use motorcycle ambulances, and about 700 missions are conducted by motorcycles each day in Tehran (the capital of Iran). The air rescue services are also used in Iran's EMS since 2000 with five helicopters. Currently there are 40 helicopters throughout the country, which shows an eight-fold increase (five helicopters) since 2005. However, existing helicopters are military ones and nonspecific for pre-hospital care. The helicopters are not owned by the emergency organization, but are provided by the police to the emergency department. Efforts are being made to purchase night-vision emergency helicopters. Five such helicopters have recently been delivered to the EMS of Iran. There are about 2600 urban and road emergency bases in the country, with 3.25 bases per 100,000 population. The first maritime emergency base was established on the southern coast of the country (Hormozgan) in 2006, and another one on the northern coast.

**Figure:**
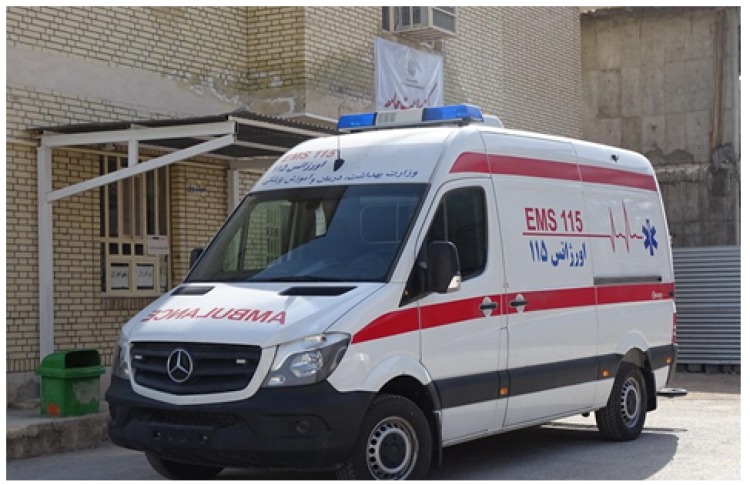
Type B ambulance in 2018 (Mercedes-Benz Sprinter 315).

**Figure:**
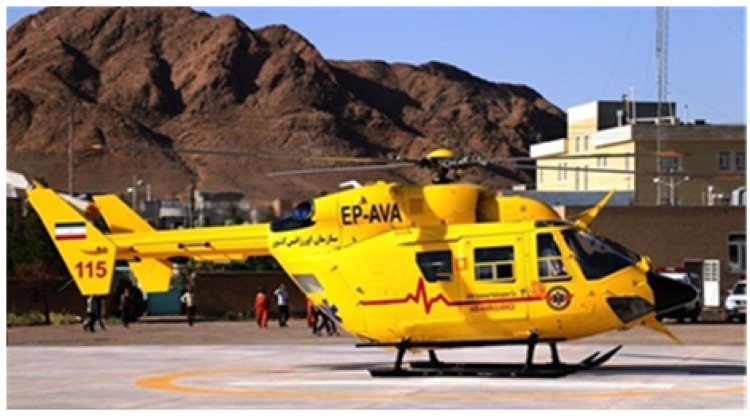
New Air Ambulance in 2018.

**Figure:**
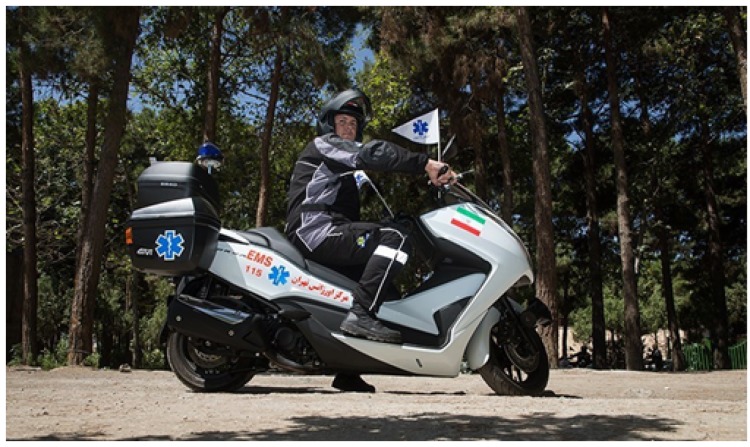
New Motorcycle ambulance in 2018 (Honda Forza SS300).

**Medical Direction**

There is a general physician serving as a medical director who is in charge of providing telephone or online radio counselling to technicians at all dispatch centers throughout the country at all times (24 hours). Since Project 247 (treating patients with acute myocardial infarction) was implemented in some cities, cardiologists are also present at the dispatch centers 24 hours a day. Technicians are required to obtain instructions from the medical director in order to take any action. There are, however, challenges in this regard, and this process is not carried out in many cases for various reasons. There are no medical directors in the districts far away from dispatch centers and rural bases, and technicians make the necessary decisions based on their own clinical judgment, experience and knowledge, which sometimes leads to legal problems for them (23). 

**New Services in EMS of Iran**

In order to achieve optimal response rate, treat patients with acute myocardial infarction, prevent the long-term complications of these patients, and reduce the cost of treatment and rehabilitation, Project 247 was implemented in some pre-hospital emergency centers, including Tabriz, Mashhad, Isfahan, Kashan, Zahedan, Lorestan, Hamedan and Tehran on July 3, 2015. The project states that: all patients with acute myocardial infarction should be transferred to hospitals and receive primary percutaneous coronary intervention (PCI) within 24 hours 7 days a week. In case of any indication, they should undergo angioplasty or coronary artery bypass surgery as soon as possible. Since December 25, 2016, the telecardiology system equipped with data-sending ability was established in EMS of Tehran. A 12-lead ECG can be taken from the patient on the accident scene and data can be sent to a cardiologist at the dispatch unit using the above-mentioned system. In case of acute myocardial infarction, the patient is immediately transferred to the intended hospital. The other project implemented since July 22, 2016 across the country is Project 724. According to this project, patients with suspected acute ischemic stroke, the symptoms of which are developed within the next 3 hours, are immediately transferred to hospitals to undergo CT scan and recombinant tissue plasminogen activator (rtPA) injections to prevent the subsequent complications. 

**Challenges**Despite significant advances in EMS in recent years, this system still faces many challenges such as human resource limitation, lack and inappropriate distribution of emergency bases, non-standard bases, ambulance burnout, lack of standard operating procedures (SOPs), imperfect medical direction, and lack of specialized fleet operations (air, rail, and marine). It is hoped that part of the problems can be solved by the government through paying special attention to this section of healthcare system by providing financial and human resources, formulation of offline protocols, implementation of a medical priority dispatch system, development of a telemedicine system, provision of dedicated helicopters, implementation of SOS, as well as educating people and increasing private sector participation.

**Conclusion and recommendation**

Historical assessment of EMS system of Iran shows that it has advanced in many different aspects. Simultaneously people’s demands have grown too, urbanization has taken place, and numbers of cars and traffic accidents have increased. Therefore, EMS needs to develop further. Until now, different EMS models from other countries have been tested and implemented in Iran, but it seems that we need to develop some context-bound model of EMS based on cultural and contextual factors. Since Iran is multicultural and the geographical situations vary in different parts of the country, we think that it is not possible to prescribe just one model for all parts of the country. It is recommended to develop specific models for each part based on the contextual, cultural and geographical factors of that part.
